# Comparison of the new fully automated extraction platform eMAG to the MagNA PURE 96 and the well-established easyMAG for detection of common human respiratory viruses

**DOI:** 10.1371/journal.pone.0211079

**Published:** 2019-02-19

**Authors:** Musa Hindiyeh, Orna Mor, Rakefet Pando, Batya Mannasse, Areej Kabat, Hadar Assraf-Zarfati, Ella Mendelson, Danit Sofer, Michal Mandelboim

**Affiliations:** 1 Central Virology Laboratory, Ministry of Health, Chaim Sheba Medical Center, Ramat-Gan, Israel; 2 Department of Epidemiology and Preventive Medicine, School of Public Health, Sackler Faculty of Medicine, Tel-Aviv University, Tel-Aviv, Israel; 3 The Israel Center for Disease Control, Israel Ministry of Health, Tel-Hashomer, Israel; Centre International de Recherche en Infectiologie, FRANCE

## Abstract

Respiratory viral infections constitute the majority of samples tested in the clinical virology laboratory during the winter season, and are mainly diagnosed using molecular assays, namely real-time PCR (qPCR). Therefore, a high-quality extraction process is critical for successful, reliable and sensitive qPCR results. Here we aimed to evaluate the performance of the newly launched eMAG compared to the fully automated MagNA PURE 96 (Roche, Germany) and to the semi-automated easyMAG (bioMerieux, France) extraction platforms. For this analysis, we assessed and compared the analytic and clinical performance of the three platforms, using 262 archived respiratory samples positive or negative to common viruses regularly examined in our laboratory (influenza A, B, H1N1pdm, Respiratory Syncytial Virus (RSV), human Metapneumovirus (hMPV), parainfluenza-3, adenovirus and negative samples). In addition, quantitated virus controls were used to determine the limit of detection of each extraction method.

In all categories tested, eMAG results were comparable to those of the easyMAG and MagNa PURE 96, highly sensitive for all viruses and over 98% clinical specificity and sensitivity for all viruses tested. Together with its high level of automation, the bioMerieux eMAG is a high-quality extraction platform enabling effective molecular analysis and is mostly suitable for medium-sized laboratories.

## Introduction

Respiratory viral infections are a major cause of morbidity and mortality worldwide [1] and have been shown to be the etiological agents of more than 70% of respiratory tract infections (RTIs) [2]. Nucleic acid-based tests, mostly performed in multiplexes by real-time PCR (qPCR) platforms, are the most common and efficient means of detecting viral infections, and support rapid and simultaneous detection of many viruses [3,4]. The nucleic acids extraction process is one of the most significant steps dictating the accuracy and sensitivity of viral infection diagnosis and virus type determination. Moreover, efficient extraction allows for the detection of different viral agents including DNA and RNA viruses, from a single extraction tube. Using qPCR technology, the Israeli Central Virology Laboratory performs viral detection on more than 10,000 respiratory samples each year, which constitute the majority of samples requiring nucleic acid extraction process in our laboratory.

NUCLISENS easyMAG (bioMérieux, France) is a second-generation silica-based semi-automatic platform that was first launched in 2005. It was specifically optimized for total nucleic acid extraction from biological samples. The system automates enhanced magnetic silica-based extraction based on the BOOM technology, a gold standard for the universal extraction of RNA and DNA based on the ability of silica to bind DNA and RNA in high salt concentrations [5].

eMAG (bioMérieux, France) is a new, fully automated nucleic acid extraction platform that enables simultaneous extraction of 48 specimens from primary tubes, either into PCR strips ready for qPCR analysis or into storage tubes. eMAG also utilizes the already well-established BOOM technology as the easyMAG [5]. MagNA PURE 96 (Roche, Germany), which was launched in 2009, was also shown to have excellent nucleic acid extraction efficiency for clinical samples [6]. This system is a fully automated extraction system that enables simultaneous extraction of 96 specimens using magnetic bead technology, from a 96 deep-well plate to a PCR plate.

The performance of the semi-automated easyMAG system has been extensively investigated; the platform was shown to efficiently extract viral nucleic acid [7,8]. In a study that compared the performance of the new eMAG and the easyMAG systems, eMAG was shown to perform equally well on several types of clinical samples including respiratory samples [9]. However, no large-scale study has simultaneously compared the performance of the eMAG to that of easyMAG and MagNA PURE 96 in nucleic acid extraction from respiratory samples.

In this study, we performed an evaluation of the newly launched eMAG as compared to both easyMAG and the fully automated MagNA PURE 96, with emphasis on the detection of influenza viruses (A, H1N1pdm09 and B), respiratory syncytial virus (RSV), parainfluenza-3, human metapneumovirus (hMPV) and adenovirus, using an in-house-developed qPCR assay [3]. Evaluations were performed using archived clinical samples collected at Sheba Medical Center in Israel, as well as calibrated controls for each virus.

## Materials and methods

### Clinical specimens and extraction platform properties

Archived nasopharyngeal samples (including Bronchoalveolar lavage (BAL) (N = 8), tracheal aspirations (N = 32) or nasopharyngeal swabs (N = 403)) of patients hospitalized at Chaim Sheba Medical Center, collected into Virocult liquid viral transport medium (LVTM) (Medical Wire & Equipment Co, Wiltshire, United Kingdom) and stored at -70°C, were used to evaluate the performance of the three extraction systems. Representative samples covering the range of cycle threshold values (Ct) below 35, which were previously extracted by easyMAG and tested for the common human respiratory viruses, were selected.

The archived samples were thawed and the volume of the sample was adjusted with M199 (transport medium Biological Industries (BI), Beit Haemek, Israel), compatible with all three systems. The archived samples (N = 262) included positive samples for influenza A (N = 47), H1N1pdm (N = 22), influenza B (N = 21), RSV (N = 21), hMPV (N = 23), parainfluenza-3 (N = 20) and adenovirus (N = 23), as well as negative control samples (N = 181).

The technical and physical properties of the three platforms, including platform size, nucleic acid collection options (tubes/plate/cartridge), sample loading method (directly/indirectly from the sample tube), the existence of a barcode reader and user operation convenience, are summarized in Table 1.

**Table 1:** A comparison between the technical properties and user operation convenience between the three platforms.

	eMAG	MagNA PURE 96	easyMAG
**Manufacturer**	bioMerieux, France	Roche, Germany	bioMerieux, France
**Properties**	fully automated	fully automated	semi-automated
**Extraction technique**	silica extraction technology (BOOM technology)	Magnetic beads technology	silica extraction technology (BOOM technology)
**Number of samples**	24+24 (48)	96	24
**Duration of extraction**	Around 90min	Around 90min	Around 60min
**Machine measurements (cm) (WxDxH)**	142x80x181	136x81.5x100	100x65x53
**Samples loading**	Samples loading directly from the original tube (1.5-14ml tubes) inside the machine	A robotic machine is needed for loading the samples (1.5-14ml tubes) into the working plate	Manual loading of the samples to the working cartridge
**Samples identification reader**	Barcode reader exists. Manual typing is very cumbersome.	Manual barcode reader exists.	Manual barcode reader exists.
**Nucleic acid collection**	Nucleic acid transferred directly to collection tubes.	Nucleic acid transferred to a collection plate. From the plate, the MagNa 96 can load the nucleic acid to a PCR plate. A robotic machine is needed for transfer to collection tubes or PCR strip.	Nucleic acid collected in the working cartridge. From this cartridge a manual transfer to the collection tubes is needed.
**User operation convenience**	Complex and cumbersome operation. The machine is composed of two independently operated subunits facing oppositely, which makes the sample tubes loading very confusing.	Very easy to operate. The sample tubes are loaded to a separate robotic machine in linear 16 samples racks. The samples plate is transferred directly to the MagNA 96.	Very easy to operate. The samples are manually loaded to the working cartridge, than placed in the Easy Mag.
**Laboratory size compatibility**	Medium-sized laboratories	Large-sized laboratories	Small-sized laboratories

### Clinical sample nucleic acid extraction and qPCR analysis

Total nucleic acids were simultaneously extracted from all patient samples, using the easyMAG, eMAG and MagNA PURE 96 instruments according to the manufacturer instructions (easyMAG and eMAG—Specific B program and in the MagNA PURE 96 -DNA and viral NA large volume kit).

Each of the samples was divided to three tubes of 500 μl each for the extraction, and extracted by the three platforms at the same time. BAL and tracheal aspiration samples were diluted with M199 transport media in case of a viscous sample. Nucleic acids were eluted into either 55μl (easyMAG and eMAG), or in 50 μl (MagNA PURE 96) elution buffer.

Multiplex qPCR reactions for detection of influenza A, B, H1N1pdm and RSV were performed as previously described [10]. In addition, multiplex qPCR was performed for the detection of hMPV, adenovirus, parainfluenza-3 and the internal control RNase P, using Ambion One-Step Real Time PCR (Life Technologies, USA). The reaction mixture contained 600nM hMPV forward and 1.2uM hMPV reverse primers, 300nM adenovirus forward and reverse primers, 300nM parainfluenza-3 forward and reverse primers, 150nM RNase P forward and reverse primers and, 200 μM of each of the assay probes. Samples from the three extraction platforms tested together by qPCR reactions for 50 cycles, using the same standard ABI 7500 instruments (Life Technology, USA).

A sample was considered positive if Ct results were under 40, regardless of the result of the internal control. All samples with Ct results above 40 were considered negative, provided that the RNase P internal control gave a positive signal (Ct<35). Invalid results could be due to qPCR inhibitors; a sample was considered invalid if no signal was obtained and the internal control was negative.

In each qPCR assay, positive internal controls for each of the targets were used. The Ct values, the standard deviation (SD) and coefficient of variation (CV) of these controls were continuously monitored. The cumulative SD and CV for each of the internal controls were always below 1SD and 2%, respectively.

### Extraction system limit of detection (LOD) for each of the targeted viruses

In order to compare the analytical sensitivity of the three extraction methods, serial decimal dilutions (10^−1^–10^−8^) of quantitated viruses including: influenza A/H3N2 (originated from world health organization (WHO)), influenza A/H1N1pdm (WHO), influenza B (WHO), RSV (American type culture collection (ATCC)), adenovirus (ATCC), hMPV and parainfluenza-3 (clinical samples), diluted in M199, were performed. These diluted viral stocks were simultaneously extracted by the three extraction systems, according to the manufacturer instructions. Eluted extracted nucleic acids were stored at -70°C until analysis. Limit of detection (LOD) was compared according to the highest detectable dilution for each sample, between all platforms using qPCR reaction.

### Data analysis

The analytical sensitivity, specificity and positive and negative predictive values (PPV and NPV) of the easyMAG and the MagNA PURE 96 were compared to those of the eMAG using Microsoft Excel 2010 software. LOD was detected when at least two of the three replicates per extracted sample were positive for the tested virus. If only one of the three triplicates was positive for a given virus, the result was considered negative (below LOD). Bland-Altman analysis was used to compare the Cts measured in the three platforms, as previously described [11].

### Ethical approval

Ethical approval for this study was granted by the Sheba Ethical Committee (approval number 4421-17-SMC), provided that anonymous samples were used. All data were fully anonymized at the time of data collection, and the committee did not require informed consent.

## Results

### Analytical parameters of the eMAG

In general, the analytical sensitivity, specificity, PPV and NPV were comparable between eMAG and the other two platforms (Table 2). The overall specificity for all tested respiratory viruses was between 98.9–100% for the easyMAG and between 97.8–100% for the MagNA PURE 96, while sensitivity ranged between 95–100% for the easyMAG and 91.3–100% for the MagNA PURE 96. PPV values of 95.2–100% were recorded for the easyMAG and 90.5–100% for the MagNA PURE 96, while the NPV values were 98.4–100% for the easyMAG and 96.8–100% for the MagNA PURE 96. Taken together, the eMAG extraction performance was comparable to that of the easyMAG and the MagNA PURE 96 (Table 2). Indeed, the kappa coefficient between eMAG and the other systems was >0.973, indicating a strong agreement between this new automated system and the other two platforms.

**Table 2:** Comparison of eMAG with EasyMAG or MagNa96.

Target virus	eMAG compared with:	No. of samples				Positive agreement		Negative agreement	
		+/+	+/-	-/+	-/-	Sens. (%)	PPV (%)	Spec. (%)	NPV (%)
**Influenza A**	EasyMAG	47	0	0	63	100	100	100	100
	MagNa96	46	1	0	63	100	97.9	98.4	100
**Influenza B**	EasyMAG	21	0	0	90	100	100	100	100
	MagNa96	21	0	0	90	100	100	100	100
**H1N1pdm**	EasyMAG	22	0	0	89	100	100	100	100
	MagNa96	21	1	0	89	100	95.5	98.9	100
**Adenovirus**	EasyMAG	22	0	1	60	95.7	100	100	98.4
	MagNa96	22	0	0	61	100	100	100	100
**RSV**	EasyMAG	20	1	1	90	95.2	95.2	98.9	98.9
	MagNa96	19	2	1	90	95	90.5	97.8	98.9
**Parainfluenza -3**	EasyMAG	19	0	1	91	95	100	100	98.9
	MagNa96	19	0	0	92	100	100	100	100
**hMPV**	EasyMAG	21	0	1	61	95.5	100	100	98.4
	MagNa96	21	0	2	60	91.3	100	100	96.8
**Overall**	**EasyMAG**	**172**	**1**	**4**	**544**	**99.4**	**97.7**	**99.3**	**99.8**
	**MagNa96**	**169**	**4**	**3**	**545**	**97.7**	**98.3**	**99.5**	**99.3**

Sens.—sensitivity; Spec.—specificity; PPV- positive predictive value; NPV-negative predictive value. The symbols +/- marks for detected /undetected.

No significant difference was found in the comparison of the performance of extraction by sample type (BAL, tracheal aspirations or nasopharyngeal swabs) with percentage agreement of above 97%.

### Intersystem comparison of cycle threshold (Ct) values

**Fig 1:**
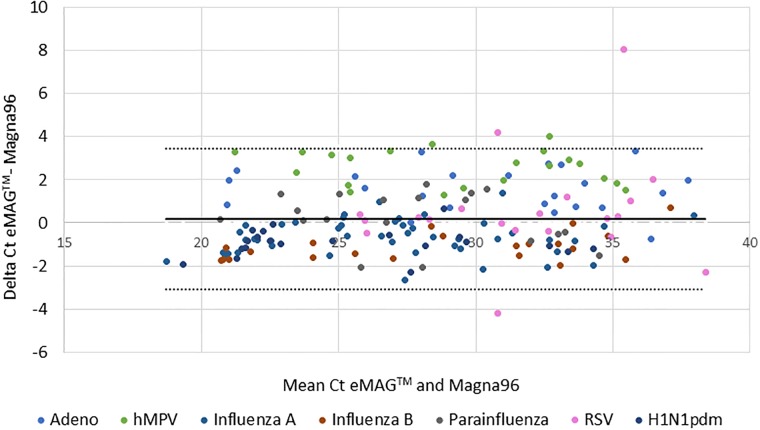
Bland-Altman analysis of the quantitative results of eMAG versus MagNA PURE 96. Bland-Altman analysis was performed on all samples for all the viruses examined, to compare the Ct values measured between eMAG and the MagNA PURE 96. The y-axis presents the delta Ct between the two platforms, while the x-axis presents the mean Ct value for both platforms for each sample. 2 SD borders are shown.

**Fig 2:**
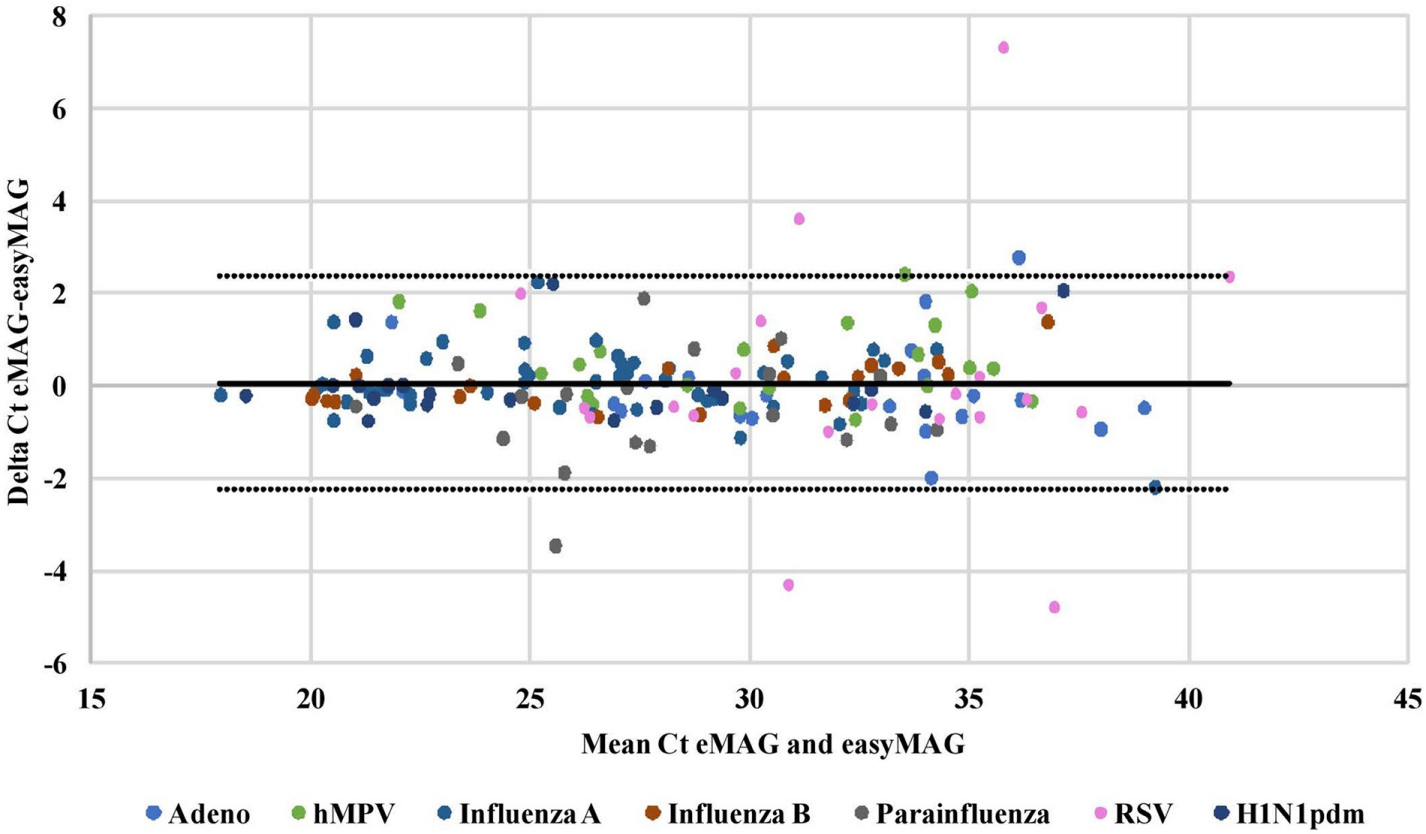
Bland-Altman analysis of the quantitative results of eMAG versus easyMAG. Bland-Altman analysis was performed on all samples for all the viruses examined, to compare the Ct values measured between eMAG and the easyMAG. The y-axis presents the delta Ct between the two platforms, while the x-axis presents the mean Ct value for both platforms for each sample. 2 SD borders are shown.

Analysis of the data revealed that the SD of the Ct values of the three platforms was less than 1.0 SD, indicating an excellent correlation between the three. Moreover, Bland-Altman analysis performed on the quantitative results obtained by eMAG versus MagNA PURE 96 (Fig 1) and between eMAG versus easyMAG (Fig 2), demonstrated similar performance. Over 98% of the samples tested in all platforms were between the SD boundaries, which indicate a good correlation between eMAG and the other two extraction methods. In both analyses, of the 21 positive RSV samples, only four samples showed different Ct values in the eMAG compared with the other two platforms.

### LOD of eMAG, easyMAG and MagNa PURE 96 for respiratory viruses

LOD was compared between the platforms using quantitated virus controls in serial decimal dilutions. We found that for most of the viruses examined (influenza B, A/H1N1pdm, adenovirus and parainfluenza-3) LOD was identical for all three systems (Table 3). For hMPV, eMAG showed a better LOD compared to the easyMAG and MagNA PURE 96, while a better LOD was provided by both eMAG and MagNA PURE 96 for influenza A, compared with the eMAG. For RSV, the easyMAG presented a better LOD than the other two platforms. Notably, all LOD differences between the three platforms resulted from a single serial dilution.

**Table 3:** Limit of detection (LOD) for each respiratory virus in all platforms.

Virus	Stock concentration (Originated from)	EasyMAG	MagNA PURE 96	eMAG
			(Dilution)	
**Influenza A**	657 copies (WHO)	10^−2^	10^−3^	10^−3^
**Influenza B**	19200 copies (WHO)	10^−3^	10^−3^	10^−3^
**H1N1pdm**	69300 copies (WHO)	10^−4^	10^−4^	10^−4^
**Adenovirus**	426000 copies (ATCC)	10^−5^	10^−5^	10^−5^
**RSV**	15800 TCID50 (ATCC)	10^−6^	10^−5^	10^−5^
**Parainfluenza-3**	Clinical sample	10^−5^	10^−5^	10^−5^
**hMPV**	Clinical sample	10^−3^	10^−3^	10^−4^

WHO- world health organization; ATCC- American type culture collection

## Discussion

In the past few years, an increasing number of respiratory clinical samples have been examined in our laboratory, the Israeli Central Virology Laboratory, Ministry of Health. As our main "gold standard", well established [12–14].extraction platform- the easyMAG can extract only 24 samples, we continuously seek to streamline the work and reduce the turnaround time. Therefore, we evaluated a larger platform- the eMAG, based on the same extraction technology as the easyMAG, which can extract up to 48 samples in each run. The eMAG was also compared with the well-validated MagNA PURE 96 that enables the extraction of 96 specimens, and currently present in our laboratory in order to evaluate the eMAG performance against another larger scale extraction platform.

First, we performed an evaluation of the analytical performance of these platforms in detecting the respiratory viruses most commonly examined in our laboratory. For most of the tested viruses, the three platforms demonstrated similar LODs. For three of the viruses, hMPV, RSV and influenza A, minor differences in LOD were noted between the platforms, all of which were within only one dilution factor. These LOD differences are negligible as these high dilution samples had very low virus concentrations and therefore are mostly undetected according to the Poisson distribution [15]. Altogether, with minor or no differences in the LOD, all three platforms presented very similar performance. As the three platforms are technically similar, especially eMAG and easyMAG, these analytical assessments were expected as previously described [9].

The clinical assessment is most important for determining the effectiveness of nucleic acid extraction and removal of enzymatic inhibitors, which have a direct impact on the qPCR results. The presented clinical assessments demonstrate a good correlation between the performance of both easyMAG and MagNA PURE 96 compared with the eMAG. Indeed, eMAG sensitivity and specificity were over 99%, compared to the easyMAG and a total of 98% sensitivity and specificity compared to the MagNA PURE 96 for all viruses tested. The clinical evaluation together with the analytical assessment allowed us to conclude that all three platforms have comparable results which allowed us to choose a larger automated extraction platform for our laboratory.

Evaluation of the technical properties of the three platforms revealed that the newly launched fully automated eMAG operation and sample loading is confusing and cumbersome when using non-barcoded tubes. Under these working conditions, the MagNa PURE 96 which is also fully automated requiring additional robotic machine, it is more user friendly compared with the eMAG. However, using the eMAG with barcoded input and output tubes enables simple load and run working mode. As for the easyMAG, it is a well-established platform and easy to operate compared with the eMAG, however, it is smaller and semi-automated (Table 1).

However, when extracting the full 48 samples without barcodes, the technical operation of this machine is a bit complex, as it is composed of two independently operated 24-sample subunits that require a well-trained technician to properly load the samples. On the other hand, these independent subunits provide an advantage when 24 or fewer samples are being tested. Assuming that the manufacturer will solve this technical difficulty, eMAG is a high-quality extraction platform mostly suitable for medium-sized laboratories.
